# Organomegaly in Mali before and after praziquantel treatment. A possible association with *Schistosoma haematobium*

**DOI:** 10.1016/j.heliyon.2017.e00440

**Published:** 2017-11-14

**Authors:** Chalotte Willemann Stecher, Henry Madsen, Shona Wilson, Moussa Sacko, Christian Wejse, Adama D. Keita, Aly Landouré, Mamadou S. Traoré, Per Kallestrup, Eskild Petersen, Birgitte Vennervald

**Affiliations:** a Department of Infectious Diseases, Aarhus University Hospital, Denmark; b Center for Global Health (GloHAU), Department of Public Health, Aarhus University, Denmark; c Section for Parasitology and Aquatic Diseases, SUND, University of Copenhagen, Denmark; d Department of Pathology, University of Cambridge, United Kingdom; e Laboratory of Parasitology, Institut National de Recerche en Sante Publique, Bamako, Mali; f University of Sciences, Techniques and Technology, Bamako, Mali; g Department of Infectious Diseases, The Royal Hospital, P.O. Box 1331, Muscat, Oman; h Institute for Clinical Medicine, University of Aarhus, Denmark

**Keywords:** Infectious disease

## Abstract

Continuous exposure to schistosome-infested water results in acute and chronic morbidity in all ages. We analysed occurence of organomegaly via ultrasonography and investigated a possible additive effect of dual-dose drug administration in 401 *Schistosoma haematobium* infected individuals from a highly endemic area in Mali. Mean intensity of infection at baseline (22.0 eggs per 10 ml) was reduced to 0.22 eggs per 10 ml 9 weeks after treatment (both treatments combined). Odds of persistent infection among those given dual-dose treatment was 41% of that in people given single dose (b = 0.41; p = 0.05; 95% CI 0.17–1.00), but after two years, 70.7% of the 157 participants, who completed the survey, were re-infected with no significant difference in prevalence and intensity of infection between treatment groups. Resolution of organomegaly occurred in all age groups after treatment. A novel association between *Schistosoma haematobium* infection and moderate portal vein enlargement was found in 35% (n: 55). Severe portal vein diameter enlargement was found in 3.2%. After two years, moderate hepatomegaly was present in 50.6%, moderate splenomegaly in 45.6% and moderate portal vein diameter enlargement in 19%. A subsequent dose of PZQ did not provide any additional long-term advantages.

## 1 Introduction

A national control program to reduce the burden of schistosomiasis infections was initiated in Mali in 1978, resulting in a national prevalence reduction from 58.9% to 26.8% of *Schistosoma haematobium*, the most prevalent schistosome species in the country [1]. Within a few years, however, prevalence rebounded to pre-intervention levels due to reinfection [1]. Programs focus on school-aged children, even though nearly half of children aged 3 months–5 years have active infections causing detrimental effects on health and development [2]. Young adults are also not included in most programs. The WHO global target of eliminating NTDs aims for a minimum of 75% coverage of preventive chemotherapy in pre-school and school-age children by 2020 [3, 4].

*S. haematobium* infection causes morbidity in the urinary tract and genital organs due to inflammation and granulomatous immunologic host responses to trapped eggs [5]. Organ lesions are positively correlated with intensity of infection [6]. Indications of genital involvement associated with *S. haematobium* infection have been shown in school aged girls [7] and untreated lesions may persist leading to reproductive and sexually related illnesses in adults [8]. Of great concern in sub-Saharan Africa is the fact, that individuals with genital schistosomiasis are at increased risk of HIV-acquisition [8, 9]. Development of hepato-splenomegaly is well known in *S. japonicum* and *S. mansoni* infections where congestive splenomegaly develops from focal inflammatory lesions provoked by eggs and dead worms resulting in necrosis, obstruction of vessels and scarring [10] but is not generally accepted as a feature of *S. haematobium* infection.

*S. haematobium* lesions resolve within the first six months after treatment, but frequently reappear within two years depending on the level of transmission [11, 12]. This has been ascribed to reinfection and a persistent low intensity infection due to the survival of young parasites, as PZQ only kills adult schistosomes [13, 14, 15, 16]. Various treatment strategies have been suggested to overcome the issue of persisting exposure such as coordinating treatment with transmission season [17] or repeating treatment when worm, that were immature at the first treatment round and thus not susceptible to PZQ, have matured [18]. PZQ has previously been shown to eradicate up to 90% of worms and eggs, but the reappearance of circulating anodic antigen (CAA) in serum as early as one week post-treatment reveals greater residual infection than previously expected [19].

This study sought to investigate a hypothesized association of organomegaly related to *S. haematobium* infection and to explore resolution after treatment in randomized treatment with single-dose or dual-dose PZQ treatment.

## 2 Materials and methods

Data collection took place in the Segou region of Mali as part of a larger multi-disciplinary study [20, 21]. The semi-arid savannah area is located 235 km from the capital, Bamako. Approximately 50% of the population lives along the Niger River. Water contact activities, such as farming, fishing, livestock keeping and daily household activities place people at risk of schistosomiasis transmission. The area is a known *S. haematobium* endemic area and malacological studies confirm that *S. mansoni* vector snails are rare in the area (Personal communication). *S. haematobium* is mainly found along rivers with no or very low prevalence of *S. mansoni*, which is found mainly in irrigation areas [22]. Demographic numbering of all houses and registration of all household members was performed by trained demographers and randomization to treatment strategy was done by computerized random selection balanced for sex and age [20, 21].

Participants enrolled in the study were between 2 and 65 years of age and had not received any previous PZQ treatment. A total of 402 individuals were screened. Sample size estimation was mainly based on comparing two proportions (proportion still positive) after single or double treatment, assuming these proportions to be 0.15 and 0.03, respectively, assuming that 80% of the participants were positive and that 15% would be lost to follow-up. Using a significance level of 0.05 and a power 90% showed that the sample size should be 175 in each group. Since only 4 people over 40 years of age participated, we limited the analysis to individuals between the ages of 2 and 40. One participant never provided urine. The residual 212 females and 189 males donated samples and were randomized into single-dose or dual-dose directly observed treatment (DOT) with PZQ 40 mg/kg body weight. A second dose of PZQ was administered two weeks after the primary treatment. The single-dose regimen was administered to 225/401 of participants (56.1%, 123 females/102 males). The second PZQ dosing was administered to 176/401 participants (43.9%, 89 females/87 males). All participants, regardless of schistosomiasis status, were treated at baseline and after two years follow-up to secure treatment of false negative individuals.

The following three samples were collected at various time points: urine, feces and blood. **Urine** was collected at baseline, 9 weeks follow-up and 24 months follow-up on three consecutive days between 10.00 and 14.00 hours. *S. haematobium* egg counts were obtained by filtration of 10 ml urine and was performed using 12 mm Nucleopore filters (Costar, Cambridge). Intensity was expressed by number of eggs per 10 ml urine. **Feces** were collected at baseline prior to PZQ treatment to assess parasitic coinfection and egg counts by Kato-Katz technique was performed twice on one stool sample. Egg counts/g feces of *Hymenolepis nana, Trichuris, Ancylostoma duodenale, Ascaris* and *Schistosoma mansoni* were calculated from a template of 41.7 mg feces by multiplying slide results with 24 as described elsewhere [22]. **Blood**. 5 ml blood in EDTA tubes was drawn pre-treatment by veni-puncture to detect for malaria parasites and IgG1- and IgG3-antibodies against *Plasmodium falciparum* for assessment of exposure by ELISA; antigen preparation and ELISA conditions are as described elsewhere [23]. Presence of malaria parasites was determined by microscopy of thick and thin smears performed by trained microscopists. Plasma was harvested and stored at −20 °C and placed on dry ice for shipment to Cambridge for antibody analysis. Samples were treated with 0.3% tributyl phosphate/1% tween for viral inactivation and subsequent storage at −80 °C [20].

**Ultrasonography (US)** was performed pre-treatment, and 9-months and 2-years post-treatment by two examiners for each participant using the consensus strategy [24]. Both examiners had extensive field experience with US examinations and followed WHO Niamey guidelines [25] using an Aloka SSD-500 3.5 MHz convex sector scanner powered by a portable generator. For liver size and portal vein diameter (PVD), the height adjustment included the linear and the quadratic term of height. For spleen length, only the linear term was needed.

### 2.1 Data analysis

Data were analysed using Stata v.14 (StataCorp LP, Texas 77845 USA). Infection status and intensity of *S. haematobium* infection were analysed in relation to gender, village, ethnic group, age groups (generated as 5 classes with approximately equal representation), treatment regimen, *S. mansoni* infection and *Plasmodium* infection using logistic and negative binomial regression analysis respectively. All factors were analysed individually and then jointly, followed by manual elimination of non-significant factors based on p-value. The models were specified as generalized linear models with a logit and log link function. Standard errors were adjusted for clustering within households. All participants who returned 1, 2 or 3 urine samples at any scheduled time (baseline, 9 weeks and 2 years post-treatment) were included in the analysis and the log_n_ of number of urine samples was used as an offset. Liver and spleen length in cm and portal vein diameter (PVD) in mm were analysed as continuous variables using linear regression as specified in a generalized linear model with a unit link function and adjusting standard errors for possible clustering in households. Predictors were the same as for prevalence and intensity of infection and the principle of analysis was the same. In addition, intensity of *S. haematobium* infection categories was added. Since the recommended WHO categories of above 50 eggs per 10 ml urine or below 50 eggs per 10 ml urine resulted in highly unbalanced size of groups we chose three groups of positive egg counts with approximately equal sample size. Negatives constituted the control group. Similarly, malaria antibody (IgG1, IgG3 and IgA) concentrations were categorized into 4 groups of equal sample sizes and used as predictors of organ sizes. Only participants, who tested positive for *S. haematobium* and took part in all 3 parasitological examinations, were included in the treatment effect analysis. Intensity of *S. haematobium* infection at baseline or during the follow-up surveys was included as predictor either as category (see above) or as continuous variable (e.g. log_n_(x + 1)). Left liver lobe length, spleen length and PVD were also assessed by scoring measurements as either normal, mildly enlarged or severely enlarged [26]. In order to assess changes in these three measures following treatment, we compared changes in measurements among those who were scored as normal, mildly enlarged or severely enlarged at baseline. Since size of left liver lobe, spleen and PVD are correlated with height [26] we worked out this relationship at baseline using polynomial regression [27]. As measurement of organ size at baseline we used the residual value. Assuming the baseline relationship between organ measurement and height at baseline would still be valid at the 9 months and 2 years follow-up, we worked out a predicted size based on height at follow-up times. Height was only measured at baseline and at the 2 years follow-up and height at 9 months was interpolated between baseline and two years height measurements considering the average age-specific height-growth rate as estimated from the height-age relationship at baseline (using polynomial regression). The difference between the actual score and the predicted mean (“residual”) was then a measure at the follow-up time. Change in organ size was the “residual” at the follow-up time minus the residual at baseline. Changes in morbidity (micro- and macro-haematuria and megaly of spleen, liver and PVD) status (present, absent) between two surveys were analysed using McNemar chi-square test (data not shown).

### 2.2 Compliance with ethical standards

The study received ethical approval from the Ethical Review Committee of the National Institute for Research in Public Health, Mali (Decision 0002/INRSP/DAP/SP- 2005). Study procedures were explained to participants prior to enrolment and oral consent was obtained from adults and parents/guardians of children prior to inclusion. Oral consent is deemed acceptable by the Malian Ministry of Health due to cultural reasons and literacy rates within rural populations.

## 3 Results

Pre-treatment, a total of 122 people (30.4%) provided all three urine samples, 222 people (55.4%) provided two samples and 57 people (14.2%) only returned one. All were included in the baseline analyses (Table 1). Overall prevalence of *S. haematobium* infection was 79.8% and prevalence was and lowest in the 2–5 years old (Table 1). Maximum intensity was 996 eggs per 10 ml urine (Table 1). When compared to the 2–5 year group, participants between 16–28 years had a 3.6 times higher odds of infection and participants in the 29–40 year group had a 1.86 times higher odds of infection. Fecal samples were collected from 291 people revealing *S. mansoni*/*S. haematobium* co-infection in 38 (13.1%). *Hymenolepis nana* was found in 9 participants (3.1%) with an egg density range of 48–8632 eggs per gram feces. Unidentified helminth eggs were found in four samples (1.4%).

**Table 1:** Prevalence and intensity of *Schistosoma haematobium* infections in people living in the Segou District, Mali. Possible predictors of these variables were tested individually (uni-variable) and then jointly, removing insignificant variables (multi-variable) using logistic and negative binomial regression, respectively.

		Prevalence^a^			Intensity		
Parameter	N	Proportion infected.	Uni-variable	Multi-variable	(Geometric mean of [eggs/10 ml] + 1) − 1	Uni-variable^b^	Multi-variable^b^
Total							
Over all	401	0.798			11.4 (996.3)		
Age group							
2–5	78	0.628	1.00	1.00	8.0 (903.7)	1.00	1.00
6–8	78	0.859	3.60**	3.50**	33.4 (996.3)	1.44	1.00
9–15	84	0.881	4.38***	3.39**	16.5 (563.0)	0.78	1.00
16–28	78	0.859	3.60**	2.55	9.1 (600.0)	0.52	0.43***
29–40	83	0.759	1.86**	1.69	4.6 (266.0)	0.24***	0.26***
Gender							
Male	189	0.820	1.00		11.8 (996.3)	1.00	
Female	212	0.778	0.77		11.1 (712.0)	0.76	
Village							
Kalabogou	216	0.704	1.00	1.00	6.0 (545.5)	1.00	1.00
Kaladagan	108	0.870	2.83***	2.73***	18.1 (996.3)	2.85***	3.03***
Guenidaga	77	0.961	10.39**	8.80**	32.7 (903.7)	3.67***	3.31***
Tribe							
Bambara	205	0.727	1.00		6.8 (545.5)	1.00	
Bozo	154	0.909	3.76***		22.2 (903.7)	2.62***	
Other	42	0.738	1.06		11.7 (996.3)	2.33	
Treatment							
Single	225	0.813	1.00		12.2 (996.3)	1.00	
Double	176	0.778	0.81		10.5 (834.5)	1.19	
*S. mansoni*							
Negative	253	0.794			10.7 (996.3)	1.00	
Positive	38	1.000	c		33.5 (834.5)	1.86*	
*S. haematobium*							
Negative	81				0.0 (0.0)		
0.3–3.5	80				1.4 (3.7)		
3.5–22.5	80				9.8 (23.0)		
22.5–90.0	80				49.0 (92.5)		
>90.0	80				234.5 (996.3)		
Malaria							
Negative	70	0.757	1.00		7.5 (766.7)	1.00	
Positive	284	0.856	1.90		14.0 (996.3)	1.46	

a coefficients are odds ratios.

b coefficients are count ratios.

c All 38 S. mansoni cases are also positive for S. haematobium; regression coefficient not estimated.

* denotes p-values < 0.05.

** denotes p-values < 0.01.

*** denotes p-values < 0.001.

Analysis of post-treatment parasitology exclusively contains participants completing all 3 parasitological examinations i.e. baseline, 9 weeks and 2 years (n = 157; 49.1%). A total of 25 people (15.9%) were still positive for *S. haematobium* eggs 9 weeks post-treatment. The final model of infection status at 9 weeks showed significance in the quadratic term of baseline intensity, i.e. baseline intensity (b = 0.43, p not significant (p ns)) and baseline intensity squared (b = 1.20, p < 0.05). In this model, females had lower odds of being *S. haematobium* positive compared to males (b = 0.23, p <0.01; 95% CI 0.09–0.62) 9 weeks post-treatment and people older than 5 years of age had increased odds of being infected than those younger than 5 (b = 8.57, p < 0.05; 95% CI 1.03–70.9). Dual-dose treatment resulted in borderline lower odds of persistent infection (b = 0.41, p = 0.05; 95% CI 0.17–1.00). After 2 years, the only significant predictor was intensity of infection at baseline (b = 1.28, p < 0.05). Participants aged 16–40 years had lower odds of infection than participants younger than 16 (b = 0.16, p < 0.001; 95% CI 0.07–0.37).

At 9 weeks, egg count predictors were the linear (b = 0.47, p ns) and quadratic (b = 1.25, p < 0.05) terms of log_n_ baseline intensity of infection. Females had lower egg counts than males (b = 0.06, p < 0.001; 95% CI 0.01–0.21) and participants older than 5 years of age had higher egg counts than participants under age 5 (b = 85.26, p < 0.001; 95% CI 11.66–623.42). Participants receiving dual-dose treatment had egg-counts that were 30% of those who received single-dose treatment (b = 0.30, p ns; 95% CI 0.05–1.68). The model, however, was over-dispersed and interpretation should be done with caution.

Size of left liver lobe, spleen length and PVD were related to height. Predictors of liver size adjusted for height were gender (females averaged 0.38 cm larger than males) and high intensity of *S. haematobium* infection (Table 2). Liver measurements were larger in people with high anti-*Plasmodium* IgG-values [IgG1-values above 325 ug/ml (b = 0.40 cm, p < 0.01) and IgG3-values above 54.9 ug/ml (b = 0.35 cm, p < 0.05)]. Due to co-linearity between the two, only IgG1-values over 325 ug/ml were retained in the multi-variable analysis, e.g. gender (b = 0.38 cm, p < 0.01), *S. haematobium* intensity category 4 (b = 0.36 cm, p < 0.01), *S. haematobium* intensity category 5 (b = 0.41 cm, p < 0.05) and IgG1-values over 325 ug/ml (b = 0.33 cm, p < 0.05).

**Table 2:** Association between size of left liver lobe, spleen length and peri-portal vein diameter and various potential predictors including *Schistosoma haematobium* infection intensity in people living in the Segou District, Mali February 2007. The factors were tested individually (“Uni-variable”) adjusting for height and possibly height squared and jointly (Multi-variable) using linear regression.

Parameter	N	Left liver lobe (cm)		Spleen length (cm)		PVD (cm)	
		N = 399; Range: 4.3–10.7		N = 398; Range: 5.4–25.0		N = 398; Range; 5.0–17.0	
		Uni-variable	Multi-variable	Uni-variable	Multi-variable	Uni-variable	Multi-variable
Height (cm)	399	0.07***^,^^a^	0.07*	0.04***^,^^a^	0.04***	−0.06^a^	−0.01
Height^2^ (cm^2^)	399	0.00**^,^^a^	0.00			0.00***^,^^a^	0.00

age group							
2–5	78	0.00		0.00		0.00	0.00
6–8	78	0.24		0.01		−0.35	0.00
9–15	84	0.34		0.07		−0.26	0.00
16–28	78	0.41		−0.07		0.07	0.57*
29–40	83	0.35		−0.64		0.55	1.05***

Gender							
Male	189	0.00	0.00	0.00	0.00	0.00	0.00
Female	212	0.38**	0.38**	−0.56**	−0.63**	−0.28	−0.40*

*S. mansoni*							
Negative	253	0.00		0.00		0.00	
Positive	38	0.18		0.83**		0.17	

*S. haematobium*							
Negative	81	0.00	0.00	0.00		0.00	
0.3–3.5	80	0.05	0.00	0.97***		0.59*	
3.5–22.5	80	0.18	0.00	0.65*		0.18	
22.5–90.0	80	0.44**	0.36**	0.71**		0.35	
>90.0	80	0.38**	0.41*	0.67**		0.47	

Malaria							
Negative	70	0.00		0.00		0.00	
Positive	284	0.09		0.46		−0.33	

Ig1							
<95	89	0.00	0.00	0.00		0.00	
95–184	89	0.25	0.00	0.36		−0.16	
185–324	89	0.20	0.00	0.51*		0.03	
325–990	89	0.56**	0.33*	1.16***	0.72*	0.32	

Ig3							
<8.8	89	0.00		0.00		0.00	
8.9–28.3	89	−0.22		0.14		−0.29	
28.6–54.8	89	0.08		0.37		0.24	
54.9–1427.3	89	0.31		0.98**	0.60*	0.08	

IgA							
<8.8	89	0.00		0.00		0.00	
8.9–28.3	89	0.06		0.24		0.02	
28.6–54.8	89	0.00		0.35		0.11	
54.9–1427.3	89	0.36		0.23		0.43	

a Coefficients are for the height and height squared as the sole predictor(s).

* denotes p-values < 0.05.

** denotes p-values < 0.01.

*** denotes p-values < 0.001.

Spleen length was linearly correlated with height. Females had relatively smaller spleens than males when keeping height constant (Table 2). Higher scores were seen in *S. mansoni* positives compared to negatives (Table 2). When adjusting for height, spleen size increased with level of anti-malarial IgG1 (< 95 ug/ml (reference category), 95–184 ug/ml (b = 0.36 cm, p ns), 185–324 ug/ml (b = 0.51 cm, p < 0.05), and 325–990 ug/ml (b = 1.16 cm, p < 0.001) and similarly for IGg3 levels, (< 8.8 ug/ml (reference category), 8.9–28.3 ug/ml (b = 0.14 cm, p ns), 28.6–54.8 ug/ml (b = 0.37 cm, p ns) and 54.9–1427 ug/ml (b = 1.16 cm, p < 0.01)). Adding these levels to the final model (Table 2) resulted in this model: height (b = 0.04 cm, p < 0.001); gender (b = -0.58, p < 0.01); *S. haematobium* intensity category 2: (0.3–3.5 eggs/g feces; b = 1.12, p < 0.001), *S. haematobium* intensity category 3: (3.5–22.5 eggs/g feces; b = 0.65, p<0.05), *S. haematobium* intensity category 4: (22.5–90.0 eggs/g feces; b = 0.73, p<0.01); *S. haematobium* intensity category 5: (>90 eggs/g feces; b = 0.65, p < 0.05); IgG1 category 4: (325–990 ug/ml; b = 0.75, p < 0.05); and IgG3 category 4: (54.9–1427.3 ug/ml; b = 0.53, p < 0.05). Portal vein diameter was larger in males than in females and in the 2 older age groups than in the younger groups. No other predictors were significant.

Liver size was classed as normal in 46.9% of participants (N = 399), while 44.1% had moderately enlarged livers and 9.0% had severely enlarged livers. Spleen size was normal in 42.6% of participants (N = 398) while 41.4% had moderate and 16.0% had severe splenomegaly. PVD was normal in 65.8% of participants (N = 398), while 30.2% had moderately and 4.0% were severely enlarged PVD.

A total of 81 participants were *S. haematobium* infection negative at baseline and excluded from the analysis of post-treatment organomegaly. Of the remaining 320 participants 236 (73.8%) were examined after 9 months. A total of 178 people (55.6%) were examined after two years.

**Fig. 1:**
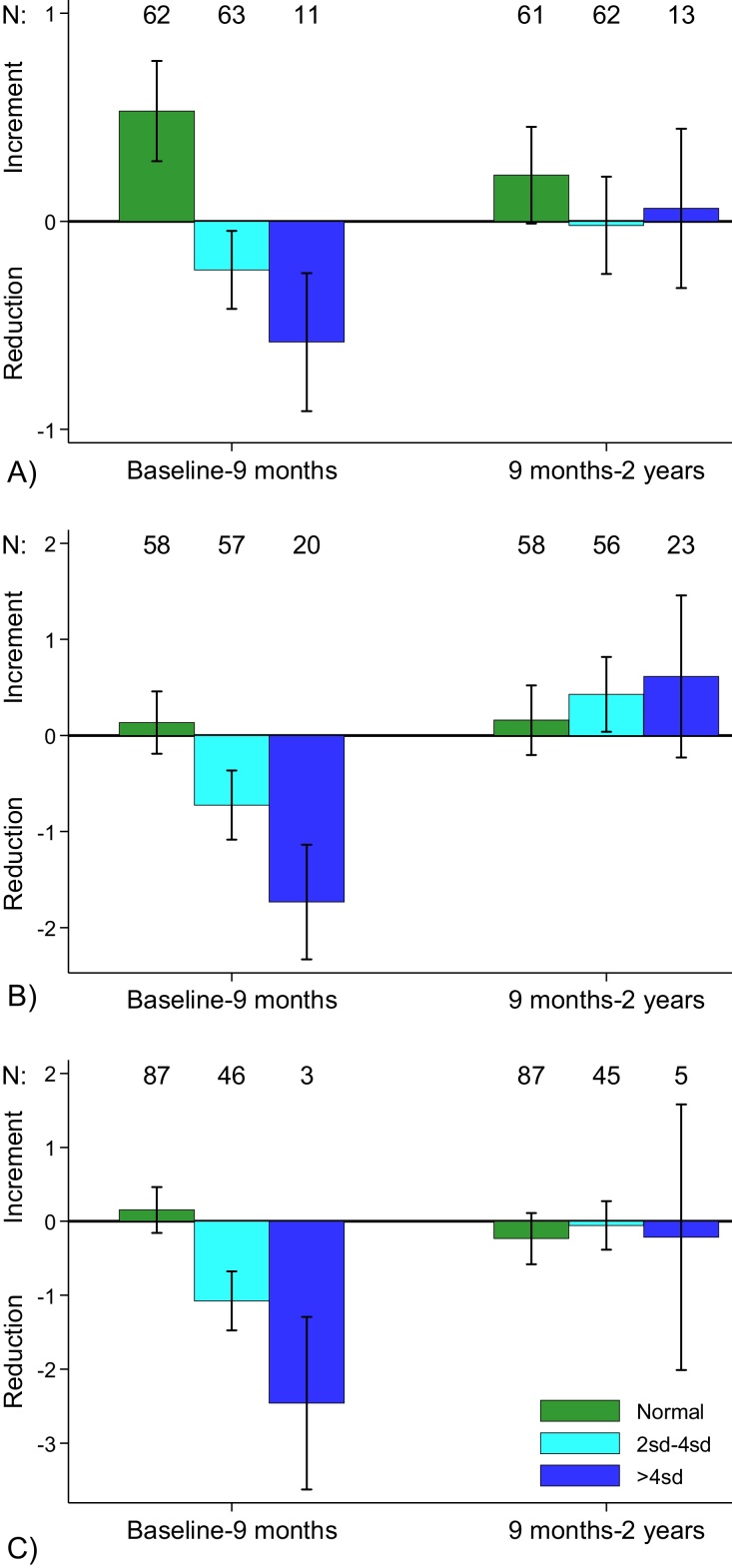
Changes in residuals (residual at the end of period minus residual at beginning of period) in liver (A) and spleen size (B) and portal vein diameter in people who a baseline were scored as normal, moderately (2sd-4sd) and severely enlarged (>4sd) based on the WHO classification.

Post-treatment data on liver-, spleen- and portal vein diameter-enlargement is presented in Table 3. Overall prevalence of organomegaly showed little effect from treatment because several cases reverted from having organomegaly to being normal and vice versa. This was true in all age groups (Table 3). Significant differences were seen when changes in organ size for height-residuals were analysed by organ enlargement classifications at baseline. Participants with normal-sized livers at baseline had averagely 0.53 cm larger livers than observed at baseline (p < 0.001), while liver size was smaller after treatment in those with moderately or severely enlarged livers at baseline: −0.23 (p < 0.05) and −0.58 (p < 0.01), respectively (Fig. 1). Significant predictors of these differences were baseline liver size and age group with the 3 older age groups having greater regression of their liver size on average (b = -0.43, p < 0.01; 95% CI −0.71–0.16) than the two younger age-groups. Participants with moderately enlarged livers (b = -0.94 cm, p < 0.001; 95% CI −1.19–0.69) and severely enlarged livers (b = -1.31 cm, p < 0.001; 95% CI −1.65–0.98) had lower scores than those with normal-sized livers (Fig. 1). Analysis of the original measurements without height adjustment showed the same trends (analysis not shown). Changes in liver size from 9 months to 2 years were small and not significantly different from zero in any of the 3 groups.

**Table 3:** Effect of treatment on prevalence and intensity of *Schistosoma haematobium* infections and prevalence of various morbidity indicators in people living in the Segou District, Mali from February 2007 (Baseline) to February 2009 (two years).

	n	Baseline	N	9 weeks	n	9 months	n	2 years
Prevalence (%)	157	100.0	157	15.9			157	70.7
Intensity (eggs 10 ml^−1^)	157	21.98	157	0.22			157	5.87
Micro-haematuria (%)	144	75.0	152	18.4			155	50.3
Macro-haematuria (%)	144	47.9	153	17.6			155	20.0
Anemia (%)	148	38.5	146	21.9			157	59.2
Bladder pathology (%)	155	11.6			139	2.9	155	27.1
Upper urinary tract (%)	155	13.5			139	7.2	155	8.4
Moderate hepatomegaly (%)	157	51.0			157	55.4	157	50.6
Severe hepatomegaly (%)	157	8.9			157	13.4	157	6.3
Moderate splenomegaly (%)	157	54.8			157	54.1	157	45.6
Severe splenomegaly (%)	157	16.6			157	15.3	157	10.8
Moderately enlarged PVD (%)	157	35.0			157	34.4	157	19.0
Severely enlarged PVD (%)	157	3.2			157	10.2	157	1.3
Mean global score	155	2.02			139	0.57	155	1.56

Spleen size in participants with normal spleens were on average 0.13 cm larger after treatment than observed at baseline (p ns), while in those with moderately or severely enlarged spleens, differences were lower, e.g. −0.72 cm (p < 0.001) and −1.73 cm (p < 0.001), respectively. The only significant predictor of changes in residuals was baseline spleen-size. i.e. for moderately enlarged (b = -0.86 cm, p < 0.001; 95% CI −1.33–0.39) and severely enlarged (b = -1.87 cm, p < 0.001; 95% CI −2.52–1.22). Changes in spleen size from 9 months to 2 years were small and not significantly different from zero in those who scored normal at baseline. Those with moderately enlarged and severely enlarged spleens at baseline had increased spleen size from 9 months to 2 years i.e. 0.61 cm, p ns; 95% CI −0.23–1.46 and 0.43, 95% CL: 0.04–0.82; p < 0.05, respectively.

PVD scores in participants with normal measurements at baseline were on average 0.16 cm larger at 9 months follow-up (p ns), while those with moderate or severe PVD enlargements at baseline had lower measurements at 9 months after treatment, i.e. −1.08 mm (p < 0.001) and −2.46 mm (p < 0.05: n = 3), respectively. Significant predictors were PVD size at baseline and age group with the three older age groups averaging lower scores (b = -0.94 mm, p < 0.001; 95CI −1.47–0.42) than the two younger age groups. In the same model, participants with moderately enlarged PVD and severely enlarged PVD at baseline had lower scores than those with normal PVDs. Changes in residuals from 9 months to 2 years were small and not significantly different from zero in any of the three groups.

## 4 Discussion

The current study conducted an ultrasonographic assessment of *S. haematobium*-related morbidity across a wide age range to detect possible organomegaly and determine the effect of two different PZQ-treatment strategies on the resolution of acute and chronic morbidity.

Our study confirmed previous findings that *S. haematobium* prevalence and infection intensity are more pronounced in the 9–15 year age group [12]. Among adults, men were more heavily infected than women and differences between villages confirm previous findings of occupational hazards for communities whose livelihood is fishing. Adding a second dose of PZQ showed a short-term benefit of lower rate of persistent infection, or acute symptoms at 9 weeks in the dual-dose treatment group, but long-term benefits for chronic lesions were not observed. Given the highly endemic environment, rapid reinfection is probable; therefore, timing of a second dose must account for reinfection and the life cycle of the parasite as young parasites are not killed and serve as a reservoir for persistent infection [28]. The stage from penetration of the human skin to the first passage of eggs via urine takes approximately 70 days [29]. The number of immature worms at any given time is highly dependent on transmission and the ideal timing of the second dose of PZQ is variable. The second dose in our study was administered after two weeks to comply with similar studies from Kenya and Uganda [30] and to secure adherence in the community.

Our study indicated an interesting reduction in the occurrence of reinfection in the 16–40 years age group, which supports the argument of including this population in treatment programs to reduce individual pathology, and also assist in breaking the transmission cycle and support resistance against possible HIV infection in this highly exposed population. Although not yet used PZQ has proven safe in small children. Studies have shown that even very young children are at risk of developing chronic lesions, which will later develop into chronic genital ulcers in adolescence and thereby enhance the risk of HIV acquisition [2]. This excluded group also plays a role in maintaining the transmission cycle of schistosomiasis and there could be an untapped chance of reducing transmission by including the youngest and oldest in MDA programs in the future.

In *S. mansoni* infected individuals, chronic hepato-splenomegaly is common and caused by egg-induced inflammation and lack of immunologic regulation [31]. Gross peri-portal fibrosis, portal hypertension, enlarged PVD, ascites and collateral vessels occur after long-term untreated exposure [32]. One study suggests a reduced risk of hepatomegaly if co-infected with *S. mansoni* and *S. haematobium* [33]. We found high prevalences of organomegaly in the community at individual levels, but pooling the overall measurements does not explain the actual dynamics and at a community level treatment does not seem to have significant impact on resolution of organomegaly because the normalized organs are replaced by diseased organs in the analysis (Table 3, Fig. 1). In the current study co-infection with *S. mansoni* occurred in few participants (13.1%), which is unlikely to explain the extension of organomegaly observed in the community. A different study from Molodo, Mali (Belonging to the Niono District, Segou region, which is an irrigation area) detects high prevalences of *S. mansoni*, but the current study has been conducted in the District of Segou, along the river Niger (Segou Region), which holds a totally different ecologic setting regarding snail habitat [22]. Ectopic abundance of *S. haematobium* infection, defined as eggs or worms trapped outside the classical genitourinary organ location, is thought to be responsible for hepatomegaly in *S. haematobium* infected individuals [33, 34]. In the current study, the most profound organ recoveries are seen during the first follow-up period after treatment and prior to the occurrence of reinfection. The demonstrated hepato-splenomegaly was most profound in participants below 6 years of age, which is also when immunity to malaria has not yet fully developed and malaria as a contribution factor to organomegaly must be taken into account. A previous study on the contribution of malaria to organomegaly in a *S. mansoni* endemic area concluded that schistosomiasis was the single most attributable factor and malaria played no role in hepatomegaly [24]. An opposing study found more serious hepato-splenomegaly in chronic malaria exposed augmented by concomitant *S. mansoni* infection and demonstrated pro-inflammatory mechanisms behind the additive effect [35]. Nevertheless, the recovery from organomegaly after PZQ in the current study cannot be explained by concomitant malaria-infection, as *Plasmodium falciparum* is refractive to PZQ treatment [36]. Distinguishing between organomegaly caused by malaria and schistosomiasis is a problem due to the geographic and seasonal overlap of the two diseases [37]. Our study demonstrated a novel finding of an association between PVD enlargement and *S. haematobium* infection intensity, which cannot be explained by malaria co-infection, but conclusively one cannot rule out the role of chronic exposure to *P. falciparum* on hepatomegaly and more research is needed to confirm the role of *S. haematobium* in development of organomegaly. Using ultrasound, PVD is an easily accessible structure to measure and a combination of the three organ-specific observations combined as an aggregated disease-score could provide a new tool for detection of schistosomiasis-related pathology.

As with all clinical studies this study has limitations. The current results correlate well with classical morbidity measures, such as intensity of infection and hematuria, but early stages of disease are not visualized by ultrasonography. One important challenge was lack of information regarding potential confounding diseases. Hepato-splenomegaly in sub-Saharan Africa is attributable to many aetiologies, such as leishmaniasis, typhoid fever, brucellosis, HIV, TB, hepatocellular cancers and chronic viral Hepatitis B and C. Additionally, 28% of individuals with pyrexia has been shown to have peri-portal thickening [37]. In the current study none of the included participants had fever, and leucocyte counts and blood pressure measurements point away from any of those chronic diseases in the population although a recent study from Mali reports a Hepatitis-B prevalence of 10–18% in adults [38]. According to the World Bank HIV-prevalence in Mali in 2015 is 1.3% in the 15–49 year age group. *S. mansoni* was found in 38 of 291 participants (13%) in our study. Almost no reinfection with *S. mansoni* was detected after treatment, suggesting that the initial *S*. *mansoni* infections were imported cases of migrating fishermen.

In conclusion we find that *S. haematobium* is a plausible cause of organomegaly in this endemic area and recommend repetition of treatment within 2 years to prevent long-term morbidity [6]. Dual-dose treatment does not reduce long-term morbidity more than single-dose regimens, mainly due to high efficiency of the first dose and unavoidable reinfection. With the global aim of eliminating schistosomiasis, MDA programs must step up and face the neglected gap of excluded populations to reduce long-term morbidity and schistosomiasis transmission and to protect against the HIV-epidemic in Africa securing the best deployment of available economic resources. Further studies, limiting the confounders of the current one, are needed to confirm the findings and extend the investigations of possible *S. haematobium* related organomegaly.

## Declarations

### Author contribution statement

Chalotte Willemann Stecher, Henry Madsen: Analyzed and interpreted the data; Wrote the paper..

Shona Wilson, Moussa Sacko, Adama D. Keita, Aly Landouré, Mamadou S. Traoré: Conceived and designed the experiments; Performed the experiments; Contributed reagents, materials, analysis tools or data.

Christian Wejse, Per Kallestrup, Eskild Petersen: Analyzed and interpreted the data.

Birgitte J Vennervald: Conceived and designed the experiments; Performed the experiments; Analyzed and interpreted the data; Contributed reagents, materials, analysis tools or data; Wrote the paper.

### Competing interest statement

The authors declare no conflicts of interest.

### Funding statement

Funding for the MaSchisMo project was provided as a scholarship from Aarhus University, Denmark. The clinical aspect is part of the MustSchistUKEMA project funded by the European Union, contract number 517733, htpp://ec.europa.eu.

### Additional information

No additional information is available for this paper.
